# Direct PtSn Alloy Formation by Pt Electrodeposition on Sn Surface

**DOI:** 10.1038/s41598-019-56749-5

**Published:** 2020-01-09

**Authors:** Jan N. Schwämmlein, Paulette A. Loichet Torres, Hubert A. Gasteiger, Hany A. El-Sayed

**Affiliations:** Chair of Technical Electrochemistry, Department of Chemistry and Catalysis Research Center, Technical University of Munich, D-85748 Garching, Germany

**Keywords:** Chemistry, Catalysis, Electrochemistry, Energy, Materials chemistry, Physical chemistry, Surface chemistry

## Abstract

Electrochemical deposition is a viable approach to develop novel catalyst structures, such as Pt thin films on conductive support materials. Most studies, reaching out to control electrochemical deposition of Pt to monolayer quantities focus on noble metal substrates (e.g., Au). In contrast, conductive oxides, such as antimony doped tin oxide (ATO), are considered as support material for different applications, e.g., as fuel cell catalysts. Herein, we investigate the deposition process of Pt on Sn, used as a model system for the electrochemical deposition of Pt on non-noble metal oxide supports. Doing so, we shade some light on the differences of a metallic Sn surface and surface oxide species in electrochemical deposition processes. With respect to a borate buffer solution, containing K_2_PtCl_4_ as Pt precursor, we report for the first time that surface oxides have the capability to fully inhibit the electrochemical deposition of Pt. Furthermore, direct alloying of the deposited Pt with the Sn support during the electrodeposition process yielded a catalyst with a high activity for the oxidation of CO.

## Introduction

Fuel cells are considered as a candidate to replace the currently wide-spread combustion engine and limit the exhaust of CO_2_, e.g., caused by automotive traffic. Especially since first car producers, such as Toyota^1^, Hyundai^2^, and Honda^3^, finally brought fuel cell powered vehicles to the market, this technology is in reach of widespread application. Nevertheless, there is a demand for novel electrocatalysts due to the low abundance of Pt and the limited stability of current catalysts under fuel cell operating conditions^4^. In this respect, film-like structures on non-noble metal based supports, such as antimony doped tin oxide (ATO), are of potential interest due to various reasons. First of all, the Pt surface to mass ratio of extended surfaces is similar to that of nanoparticles for sufficiently thin layers. Second, Pt thin film structures may show high specific activity towards the ORR due to a likely exposure of low index facets, providing a superior ORR exchange current density compared to Pt nanoparticles^5^. Third, a contiguous film may protect the underlying support material against corrosion. Finally, conductive oxides were proposed as stable catalysts with respect to high anodic potentials, occurring under certain fuel cell operating conditions, such as start-up or shut-down (SUSD)^6–8^.

In contrast to the lack of synthesis methods for Pt thin films on non-noble metal oxides, various methods were already presented to generate low loading platinum deposits on metallic supports. One approach was to utilize the redox replacement of an underpotentially adsorbed Cu layer by Pt from a precursor dissolved in the electrolyte^9^. Another method, developed by Brimaud *et al*. utilizes the strong adsorption of CO on the deposited Pt to hinder any further deposition after completion of a single Pt monolayer (ML) on a Au(111) single crystal^10^. Though the Pt ML was reported to be stable while CO was adsorbed, the group showed that the structure collapsed to Pt islands as soon as CO was displaced. A similar effect to limit Pt electrodeposition on Au(111) to an amount of Pt equivalent to one ML was presented by Moffat and co-workers^11^. Instead of CO, the potential dependent formation of H_ads_ was used to interrupt the deposition process while the hydrogen evolution reaction (HER) proceeded on the Pt deposit. To avoid the formation of H_2_ bubbles, the group used an unbuffered electrolyte with a pH of 4, where the amount of HER was limited due to an evolving pH gradient between the electrode and the bulk of the electrolyte^12^. The scanning tunneling microscopy (STM) images of the resulting deposits showed an island-like morphology of very small Pt deposits on the Au surface. Similar to those studies, most electrochemical deposition techniques, aiming to achieve low loading Pt deposits focus on noble metal substrates, whereas deposition of low platinum quantities on non-noble metals or conductive oxides was only scarcely reported (e.g., on Ni)^13^.

In this publication, we present insights into the electrodeposition process of Pt on Sn as a model system for such novel catalysts. Overall, this manuscript aims to deliver a better understanding of the electrodeposition process, eventually leading to a synthesis approach for novel fuel cell catalysts, comprised of low Pt loaded conductive oxides. At first, the electrochemical behavior of Sn in slightly alkaline solution is investigated to identify the oxidation and reduction processes on its surface. Subsequently, the influence of oxides on the electrochemical deposition process of Pt on the Sn surface is evaluated. Finally, Pt deposition is carried out in the presence of HER, where the resulting Pt film thickness is studied by energy dispersive X-ray (EDX) spectroscopy. The electrochemical oxidation of CO is used as a probe for the structure of the deposits. By this method, we are able to identify the deposits as PtSn alloys, formed directly on the surface of the electrode during the deposition process.

## Methods

### Rotating disk electrode (RDE) setup

A home-made glass cell was used for all electrochemical measurements presented here. All compartments of the cell were thoroughly cleaned in Caro’s acid and heated repetitively in fresh, ultrapure water (18.2 MΩ cm, MilliQ Ingegral, *Merck Millipore KGaA*, Germany) to eliminate sources of contamination. Borate buffer (0.05 M) was prepared from high purity H_3_BO_3_ (99.97%, trace metals basis, *Sigma Aldrich Corp*., Germany) and NaOH ∙ H_2_O (99.9995%, metals basis, TraceSELECT, *Sigma Aldrich Corp*., Germany) by addition of ultrapure water. The pH was measured with a pH meter by separating a small portion of the electrolyte and subsequently adding sodium hydroxide or boric acid to the stock solution until the desired value of 8.4 was reached. Argon used to saturate the electrolyte was of high purity (6.0 grade, *Westfalen AG*, Germany), as well as carbon monoxide (4.7-grade, *Westfalen AG*, Germany) for CO oxidation voltammetry. A home-made Ag/AgCl reference electrode, saturated with KCl (99.999%, *Sigma Aldrich Corp*., Germany) was used for all measurements. The reference potential of the Ag/AgCl electrode was calibrated with the platinum ring of the electrode by saturating the electrolyte with H_2_ prior to the performed experiment. The reference electrode was separated from the cell compartment by an electrolyte bridge to avoid contamination of the electrolyte with chloride ions. All potentials in this publication are reported vs the reversible hydrogen electrode (RHE) potential, calculated by subtraction of the reference electrode potential from the measured value. All area-normalized currents refer to the geometric area of the electrode (e.g., cm^2^), if not otherwise stated. Electrochemical measurements were performed using an Autolab potentiostat (PGSTAT302N, *Metrohm AG*, Switzerland) and a rotator (*Pine Research Instrumentation*, USA) with a polyether ether ketone shaft.

### Electrode preparation

Polycrystalline Sn electrodes (99.999%, *MaTecK Material-Technologie & Kristalle GmbH*, Germany) were prepared by polishing in three individual steps using a 9 µm diamond suspension (MetaDi Supreme) on a VerduTex polishing cloth, a 3 µm diamond suspension (MetaDi Supreme) on a MicroCloth and 1 µm Al_2_O_3_ (MicroPolish II) on a Microcloth with a polishing machine (MetaServ 250/vector head), all purchased at *Bühler GmbH* (Germany). Each polishing step was performed for at least 5 minutes and the crystal was sonicated at least five times in ultrapure water before moving on to the next step, mitigating Pt contamination on the Sn crystals.

### Electrochemical deposition procedure

Potential-controlled electrochemical deposition (−0.10, −0.15, −0.25 V_RHE_) was carried out by introducing K_2_PtCl_4_ (99.999%, *Sigma Aldrich Corp*., Germany) to the Ar-saturated electrolyte while the electrode was rotated at 200 rpm. The Pt precursor was dissolved in borate buffer (1 mL) prior to addition and the amount of K_2_PtCl_4_, added to the cell, was adjusted to yield an overall concentration of 1.5 mM. After the desired time of deposition was reached, the electrode was immediately removed from the solution and rinsed with ultrapure water to remove any remaining Pt ions from the surface of the electrode.

### Pt sputtering and scanning electron microscopy (SEM)

Platinum was sputtered on polished Sn samples in Ar atmosphere (0.05 mbar), using a sputtering machine (Sputter Coater SCD 004, *Fluke Corp*., USA) with a working distance of 5 cm at an applied current of 15, 45, 60, 30 or 60 A for 40, 30, 26, 180 or 52 s to achieve a Pt overlayer thickness of 5, 20, 40, 60 or 80 nm. Images of the deposits were taken with a high resolution scanning electron microscope (JCM-7500 F, *Jeol Germany GmbH*, Germany) after washing the samples with ultrapure water and drying them for at least 24 h at room temperature. EDX spectroscopy was carried out on a table top SEM-EDX device (JCM-6000, *Jeol Germany GmbH*, Germany) at 15 kV.

### XPS characterization

The XPS measurements were performed in a Kratos Axis Supra spectrometer with a monochromatic Al Kα X-ray source. The PtSn samples were mounted non-conductive in the sample holder by using a carbon tape and a PTFE sheet to adjust the sample’s height. The binding energies for each spectra were corrected using the C 1 s peak (284.6 eV) as a reference. The peak areas from the Pt 4 f and Sn 3d level were calculated with casaXPS processing software. A Shirley background was employed for the background subtraction during the area calculation.

## Results and Discussion

### Electrochemistry of Sn

**Figure 1:**
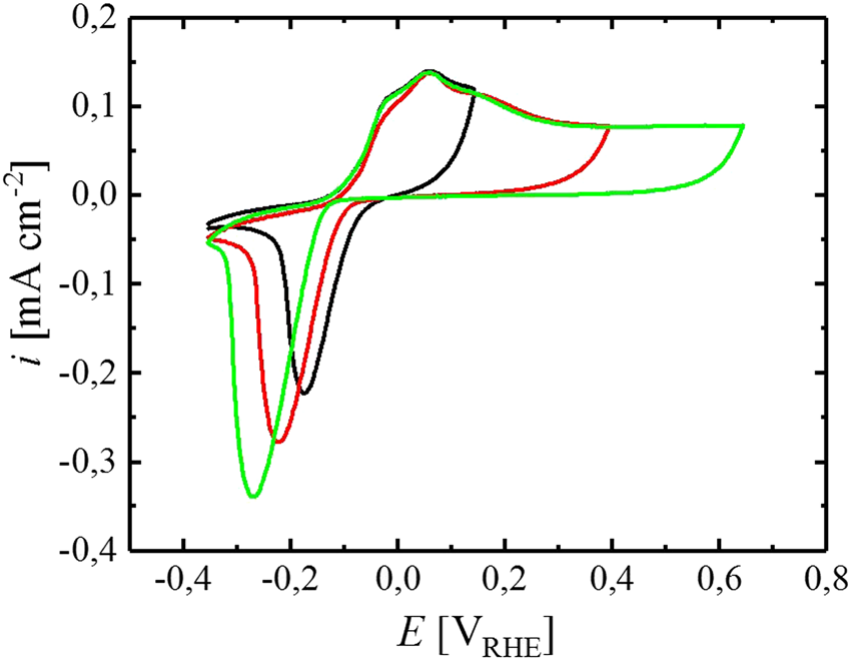
CVs of a Sn electrode in Ar-saturated borate buffer (0.05 M, pH = 8.4) at a scan rate of 20 mV s^-1^ between −0.35 and 0.14 (black), 0.39 (red) or 0.64 V_RHE_ (green) without rotation, measured at room temperature. Origin 9.1 (https://www.originlab.com).

As a non-noble metal, Sn readily forms an oxide on its surface if exposed to an oxidizing agent, such as O_2_ in air. Furthermore, Sn dissolves to Sn(II) and Sn(IV) species in acidic and alkaline media^14^. In contrast to this, metallic Sn is thermodynamically stable between approximately −0.1 and −1.0 V_RHE_ in solutions with neutral pH^15^. Therefore, Sn can be reversibly oxidized and reduced by potential control in neutral media, e.g., without significant dissolution into the electrolyte^16^. Accordingly, the steady-state CV of a Sn electrode in a borate buffer solution with a pH of 8.4 is presented in Fig. 1, showing oxidative features at potentials more anodic than −0.1 V_RHE_, as well as reductive features at more cathodic potentials. The electrochemical behavior of Sn has been studied in various electrolytes by different groups^16–19^, and the following reaction scheme was proposed for the electrochemical oxidation of metallic Sn in borate buffer by Kapusta *et al*.^16^.

1 $${\rm{Sn}}+2\,{{\rm{OH}}}^{-}\to {\rm{Sn}}{({\rm{OH}})}_{2}+2\,{{\rm{e}}}^{-}$$

2 $${\rm{Sn}}{({\rm{OH}})}_{2}+2\,{{\rm{OH}}}^{-}\to {\rm{Sn}}{({\rm{OH}})}_{4}+2\,{{\rm{e}}}^{-}$$

In accordance with these reports, the anodic current shown in Fig. 1 is assigned to the oxidation of the Sn surface, whereas the cathodic peak is ascribed to the associated reduction to metallic Sn. In the absence of a continuous reaction (e.g., HER), the extent of reversibility of the redox process can be estimated by dividing the integral of the reductive charge (*C*_red_) by the oxidative charge (*C*_ox_), passing through the electrode during a single cycle. Considering the CV with an anodic potential limited of 0.14 V_RHE_ (Fig. 1, black line), the oxidative and cathodic charges are perfectly balanced (*C*_red_/*C*_ox_ ≈ 1), hence the process of surface oxide formation on Sn can be considered fully reversible within this potential range. When the anodic vertex of the CV is increased to higher potentials (0.39 and 0.64 V_RHE_), the oxidative as well as the reductive charge increases and the cathodic peak shifts to more negative potentials (Fig. 1, red and green lines). This observation can be explained by the transformation of the anodically formed oxide to a more stable oxide layer on the Sn surface at these higher anodic potentials, leading to an increase in the reduction overpotential in the negative going scan^16^. Furthermore, the charge balance *C*_red_/*C*_ox_ decreases to ≈0.7 and ≈0.6 when the anodic vertex increases to 0.39 and 0.64 V_RHE_, respectively. The higher oxidative compared to reductive charge indicates an incomplete reduction of the oxide layer within the applied potential region, most likely due to the anodic stabilization of the surface oxide. In alignment with this hypothesis, Kaputsa *et al*. reported that full removal of certain, anodically grown Sn oxide species requires extended periods at substantially more cathodic potentials, including the formation of H_2_ on the electrode^16^.

### The role of surface oxides in the electrodeposition of Pt

**Figure 2:**
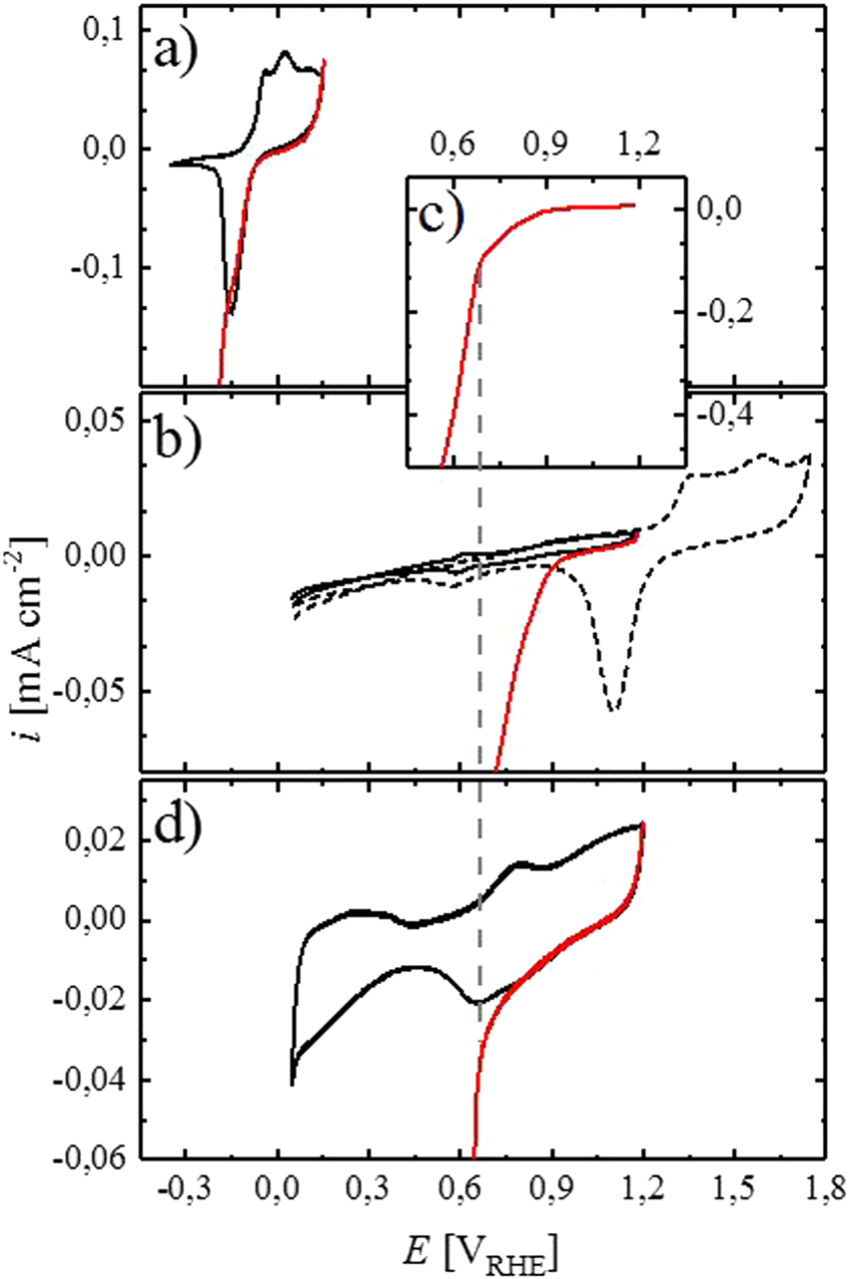
The solid, black lines show CVs of a) Sn (−0.35–0.14 V_RHE_), b) Au (0.05–1.18 V_RHE_) with a zoomed inset in c) and d) Pt (0.05–1.20 V_RHE_) in Ar-saturated borate buffer (0.05 M, pH = 8.4) at a scan rate of 10 mV s^−1^, applying a rotation rate of 400 rpm, measured at room temperature. At the anodic vertex of the CV, a solution containing K_2_PtCl_4_ (1.5 mM) was added to the electrolyte, with the red lines showing the cathodic scan directly after addition of the Pt precursor. The dotted, black line in b) shows a CV of a Au electrode at more anodic potentials (0.05–1.75 V_RHE_), as a comparison. The dashed, grey line is solely a guide for the eye to indicate the change of slope in the deposition transient. Origin 9.1 (https://www.originlab.com).

In general, the deposition of metals on oxides is associated with a large interfacial energy, often leading to the formation of islands due to a dewetting effect^20^. The effect of Sn oxide on the electrochemical deposition of Pt was studied here using CV in borate buffer solution. Pt deposition on Sn was carried out after potential cycling of the electrode surface in the potential range of the fully reversible oxide formation (i.e., between −0.35 and 0.14 V_RHE_) until a stable voltammogram was established, whereas high anodic potentials were avoided to abstain from the irreversible oxide formation. When the CV of the Sn electrode reached the anodic vertex of 0.14 V_RHE_, an already dissolved Pt(II) precursor (K_2_PtCl_4_) was added to the electrolyte and the potential was scanned in cathodic direction. Hence, an oxide is present on the surface of the electrode when the precursor is introduced to the system. Figure 2a shows the CV prior to addition of the Pt precursor (black line) and the subsequent cathodic scan in the presence of Pt ions (red line). With respect to thermodynamics, facile Pt deposition on the electrode surface is expected in the entire applied potential region, due to the large cathodic overpotential of more than 1 V with respect to the standard reduction potential of PtCl_4_^2−^ (0.755 V_SHE_) according to the following reaction scheme^21^.

3 $${{\rm{PtCl}}}_{4}^{2-}+2{{\rm{e}}}^{-}\to {\rm{Pt}}+4{{\rm{Cl}}}^{-}$$

Nevertheless, as shown (red line) in Fig. 2a, the current in the cathodic sweep after the addition of the Pt precursor initially follows the previous cycle, where no PtCl_4_^2−^ ions were present in the electrolyte. The absence of a cathodic current in addition to that originating from the redox process on the Sn electrode indicates that Pt deposition from a borate buffer solution does not occur on the Sn electrode between 0.14 and ≈−0.12 V_RHE_. Nevertheless, the current increases significantly when the potential is scanned further cathodic into the range corresponding to the reduction of Sn oxide species, indicating the onset of Pt deposition. Sweeping the potential further cathodic leads to a linear increase of the current, accounting for ongoing reduction of Pt ions from solution and HER on the resulting deposits. According to these findings, we hypothesize that Pt deposition on Sn is not possible under the applied conditions as long as the metallic surface is covered with oxide species. It can be excluded that the observed phenomenon is due to a time transient effect, where the Pt precursor would not be present at the surface of the electrode until the increase of the current is observed, since the electrode was rotated (400 rpm) during the experiment, ensuring a quick transport of the already dissolved precursor to the electrode surface. Furthermore, similar experiments were performed at different potentials (shown at a later point of the study, Fig. 4) where the insertion of the precursor led to an instant rise of the cathodic current. In order to verify that surface oxides hinder the electrochemical deposition process on Sn, a similar experiment was carried out on a Au electrode. Compared to Sn, the electrochemical oxidation of Au in borate buffer takes place at substantially more anodic potentials >1.2 V_RHE_ (dashed, black line, Fig. 2b). Limiting the anodic vertex of the CV to a potential before the oxidation of Au ensures the metallic state of the electrode, hence no reductive peaks appear in the cathodic scan of the CV (solid, black line, Fig. 2b). By adding the Pt precursor at 1.2 V_RHE_ and scanning the potential cathodic, the onset of Pt deposition on Au was found at ≈0.9 V_RHE_ (red line, Fig. 2b). Owing to the fact that this potential lies more than 1 V anodic of that found on Sn, we conclude that Pt deposition on Sn is indeed fully prevented in the presence of an oxide layer on the surface. Continuing the cathodic sweep reveals a change of slope with respect to the increase in the deposition current at a potential of approximately 0.66 V_RHE_, best visible in Fig. 2c. The charge that passed the electrode in the cathodic scan before reaching 0.66 V_RHE_, is approximately 720 µC cm^−2^. Considering a 2e^−^ process for the reduction of PtCl_4_^2−^ according to Equation [3], and assuming 100% efficiency, this charge corresponds approximately to the deposition of one Pt monolayer on Au (assuming a hypothetical roughness factor of ≈1.5 ${{\rm{c}}{\rm{m}}}_{{\rm{p}}{\rm{t}}}^{2}{\rm{c}}{\rm{m}}$^−2^ for the Au disk). Hence, the majority of the Au surface is already covered with Pt when the potential of 0.66 V_RHE_ is reached. Repeating the deposition experiment on a Pt electrode (Fig. 2d) gives further insight into the Pt deposition process. Similar to Sn, deposition of Pt on Pt starts after the removal of surface oxides on the metal (red line, Fig. 2d). The onset of Pt deposition on the Pt disk was ≈0.66 V_RHE_, coinciding well with the potential where a change of slope was observed when Pt is deposited on the Au disk (red line, Fig. 2b). Hence, we hypothesize that Pt deposited on Au at potentials more anodic than 0.66 V_RHE_ is oxidized quickly, forming a layer of oxide species. This layer may hinder the continuation of the deposition process, similar to that on a Pt (or Sn) electrode and might eventually stop the process entirely in a certain potential region. By sweeping the potential further cathodic, metallic Pt is exposed and Pt deposition can continue unabated, leading to an increase in the deposition current, thus a change of slope in the cathodic scan of Fig. 2b is observed. According to the results presented here, we propose that oxide species on the surface of Pt and Sn electrodes are capable of fully inhibiting Pt deposition under the conditions applied here, even though high cathodic overpotentials are applied to the system. As mentioned earlier, similar self-limiting mechanisms with respect to the deposition of Pt were observed by other researchers who utilized this effect to prepare Pt-ML structures on Au. Brimaud *et al*. deposited Pt electrochemically on Au in the presence of CO in the electrolyte. They reported that the rapid adsorption of carbon monoxide on the Pt surface fully blocks further electrodeposition^10^. Liu *et al*. employed a similar method to obtain a Pt-ML on Au, where they used the underpotential deposition of hydrogen (H_upd_) to stop the Pt deposition after the formation of the first layer^11^. If developed further, the mechanism reported in this publication could potentially be utilized in a similar way to obtain a Pt-ML or thin film on a non-noble metal support.

**Figure 3:**
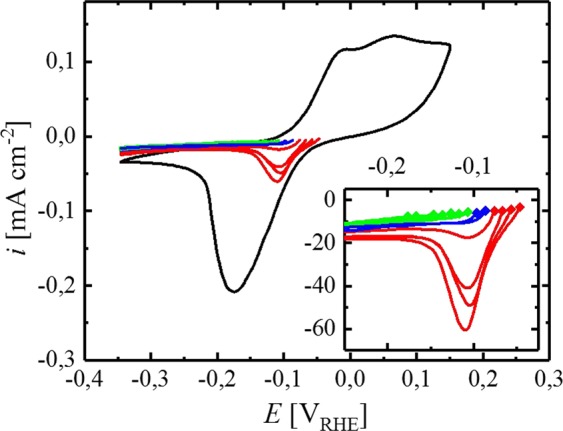
Steady state CV (black line) of a Sn electrode in Ar-saturated borate buffer (0.05 M, pH = 8.4) at a scan rate of 20 mV s^−1^ between −0.35 and 0.14 V_RHE_, measured at room temperature. Red, blue and green lines show linear potential scans in cathodic direction at 20 mV s^−1^ after a constant potential was applied for 120 s. The applied potential is indicated by a diamond, which can be seen better in the inset, which shows a zoom of the same graph. Origin 9.1 (https://www.originlab.com).

Since Pt deposits only on metallic Sn, an experiment was designed to identify the potential at which Sn oxide formation takes place on the surface of the electrode. First of all, a CV, terminating at a potential of −0.35 V_RHE_, was carried out to reduce the surface oxide of the Sn electrode. Thereafter, the potential of interest was applied for 2 minutes (chronoamperometric period) to either stabilize the surface in the metallic state or to form surface oxide species. Subsequently, the potential was scanned cathodic to probe for the appearance of a peak, corresponding to the reduction of the surface oxide to metallic Sn. When potentials more negative than −0.1 V_RHE_ are applied during the chronoamperometric period, no peak appears in the cathodic scan (green lines, Fig. 3), thus no oxidation of the metallic surface has taken place and the probed potential is considered suitable for the electrodeposition of Pt. In contrast to that, clear reduction features are observed after applying potentials more positive than −0.09 V_RHE_ (red lines, Fig. 3) due to (partial) oxidation of the Sn surface. In the transition region between those two potential regions, no clear peak is found but slight reoxidation of the surface cannot be fully excluded (blue lines, Fig. 3).

**Figure 4:**
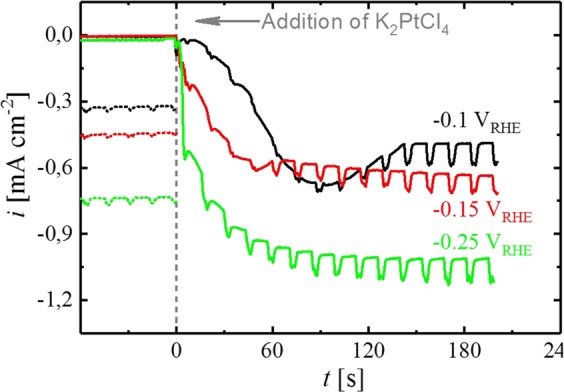
Current transient of potentiostatic measurements on Sn electrodes at −0.1 (black), −0.15 (red) and −0.25 V_RHE_ (green) in deaerated borate buffer (0.05 M, pH = 8.4) at a rotation rate of 200 rpm, interrupted by a 3 s rotational pulse of 3000 rpm every 15 s, measured at room temperature. A borate buffer solution, containing K_2_PtCl_4_, was added to the electrolyte (time of addition depicted by a dotted, grey line), resulting in an overall concentration of 1.5 mM. Origin 9.1 (https://www.originlab.com).

### The nature of electrodeposited Pt on Sn

In order to obtain an insight into the deposition process, as well as into the morphology of the resulting Pt deposits, potentiostatic electrodeposition was carried out at different potentials and the deposits were imaged by SEM. Furthermore, the change in the electrochemical properties of the Pt deposits, as well as the increase in the film thickness throughout the deposition process with respect to the deposition time are addressed in this section.

To ensure the metallic state of Sn prior to the electrodeposition process, potentiostatic Pt deposition was solely carried out at sufficiently cathodic potentials, ensuring the absence of oxide species on Sn according to Fig. 4, viz., −0.1, −0.15 and −0.25 V_RHE_. Prior to deposition, the Sn oxide on the electrode surface was reduced by scanning the potential cathodic to −0.35 V_RHE_, followed by a stabilization period at the respective deposition potential for 120 s (in the absence of Pt ions in the solution). Thereafter, K_2_PtCl_4_ pre-dissolved in borate buffer was added to the solution to initiate Pt deposition on Sn. During the electrochemical deposition process, a rotation rate of 200 rpm was applied, interrupted every 15 s by a rotational pulse of 3000 rpm for 3 s to remove hydrogen bubbles from the surface of the electrode.

As shown in Fig. 4 (solid lines), the current at each potential remains constant before the addition of the Pt precursor, indicating steady conditions and the absence of additional reductive processes, e.g., due to the removal of residual surface oxides. As soon as K_2_PtCl_4_ is introduced to the electrolyte, the current increases in all experiments, eventually sloping out to a plateau for the remainder of the potentiostatic period. Therefore, the current increase can clearly be ascribed to the reduction of Pt ions at the surface of the electrode, superimposed with a co-current from HER on the Pt deposits. In contrast to Sn^22^, Pt is highly active for HOR/HER in a wide range of pH values^23–27^. Therefore, a significant increase of the current is expected for a Sn electrode once it is at least partially covered with Pt due to the large applied HER overpotential. Consistently, the cathodic current after the addition of the Pt precursor originates from Pt deposition, as well as from HER on the Pt deposits. The application of a larger HER overpotential (more cathodic potential) therefore causes a higher current during the plateau phase. Moreover, the time needed to reach the plateau is clearly dependent on the applied potential, with more cathodic potentials enhancing the rate of the deposition process. As a result, the Sn surface is more quickly covered with Pt at more cathodic potentials and the plateau, corresponding to the maximum rate of HER, is reached more quickly. Consistently, the current measured at −0.1 V_RHE_ (Fig. 4, black line) requires significantly more time to reach steady state and reproducibly shows a current peak before the final plateau. We account this to an overlap of Pt deposition on uncovered Sn sites with the current originating from HER on Pt, since covering the electrode with Pt requires more time compared to −0.15 (Fig. 4, red line) and −0.25 V_RHE_ (Fig. 4, green line). However, according to Strmcnik *et al*., the surface of Pt is fully covered with H_ads_ in the relevant potential region (≤−0.1 V_RHE_)^28^. Liu *et al*. utilized the rapid formation of Pt-H_ads_ on the surface of the deposited Pt to prepare a Pt-ML on a single crystalline gold surface, where Pt electrodeposition was fully prevented by the adsorbed hydrogen^11^. Since the potential range utilized for deposition experiments in this study is similar to that of Liu *et al*. (−0.33 V_RHE_), one might expect a comparable inhibition of the deposition process on Sn. However, the electrolyte used by Liu *et al*. was an unbuffered NaCl solution (0.5 M), where the pH at the surface of the electrode shifts due to the consumption of H^+^ during HER on Pt^12^. The advantage of this approach is the possibility to adjust the rate of H_2_ evolution to a moderate level by choosing the solution pH accordingly. Hence, using an electrolyte with a pH of 4 (according to Liu *et al*.) results in an HER limiting current of ≈100 µA cm^−2^ at a rotation rate of 400 rpm^11^. In this case, the associated molar flux of H_2_ produced at the surface of the electrode is as low as ≈0.5 nmol s^−1^ cm^−2^ (calculated via Faraday’s law at a current density of 100 µA cm^−2^ in a 2 e^−^ transfer reaction). In contrast to this rather low H_2_ production rate, the maximum flux transported away from the electrode through diffusion under the applied conditions is on the order of 15 nmol s^−1^ cm^−2^ (calculated via Fick’s first law, assuming a diffusion boundary layer thickness of 40 µm at 400 rpm, a maximum H_2_ concentration of 1.3 mM at 25 °C^25^, a diffusion coefficient of 4.5 ∙ 10^−9^ m^2^ s^−1^ and the absence of H_2_ in the bulk of the solution)^29^, allowing the produced H_2_ to be easily transported away from the electrode into the bulk of solution without forming bubbles on the electrode surface. In accordance with this, a solution pH of 8.4, i.e., as used in all Pt deposition experiments in this article, results in a theoretical limiting current of 12 nA cm^−2^ (calculated via the Levich equation using a diffusion coefficient of 7 ∙ 10^−9^ m^2^ s^−1^ and a rotation rate of 400 rpm)^30^ due to the comparably low proton concentration. However, in unbuffered electrolytes, the concentration of protons at the surface of the electrode depletes when HER proceeds, resulting in a shift of the surface pH. Hence, due to the limited stability window of Sn with respect to pH (Sn dissolves in acidic and alkaline media)^15^, a buffered electrolyte was used in all experiments described here. Since protons can be readily replenished in buffers, a change of the solution pH is avoided and the H^+^ concentration in the vicinity of the electrode does not deplete analogously to an unbuffered electrolyte. Therefore, the current observed on a polycrystalline Pt disk (Fig. 4, dotted lines) in the same electrolyte, at the potentials used for electrochemical deposition, i.e., −0.10, −0.15 and −0.25 V_RHE_, ranges between 0.34–0.76 mA cm^−2^ at a rotation rate of 400 rpm. Hence, the rate of H_2_ evolution at the surface of the electrode is approximately one order of magnitude larger compared to Liu *et al*. Accordingly, H_2_ bubbles can form on the surface of the electrode, eventually being released into the electrolyte. The surface sites liberated in this process may serve as template for further Pt deposition, hence the deposition process is not expected to be fully inhibited under the conditions applied here. Nevertheless, it is expected that the rate of Pt deposition is significantly decreased due to blockage of Pt sites by adsorbed hydrogen. In principle, a rough estimation of the efficiency of the deposition process during the plateau phase can be made by subtraction of the charge originating from HER on pure Pt from the charge obtained during the electrodeposition experiments. Dividing this “HER-corrected” charge by the total charge delivered during the same period of time yields a maximum deposition efficiency of 30, 23 and 24% at −0.10, −0.15 and −0.25 V_RHE_. Even though an extraction of the amount of deposited Pt by this method is rather delicate due to differences in the nature of the electrode (Pt deposits on Sn versus pure Pt), the electrode morphology, and the additional salt in the electrolyte, the low calculated efficiency indicates that the deposition of Pt on the electrodes is strongly limited.

**Figure 5:**
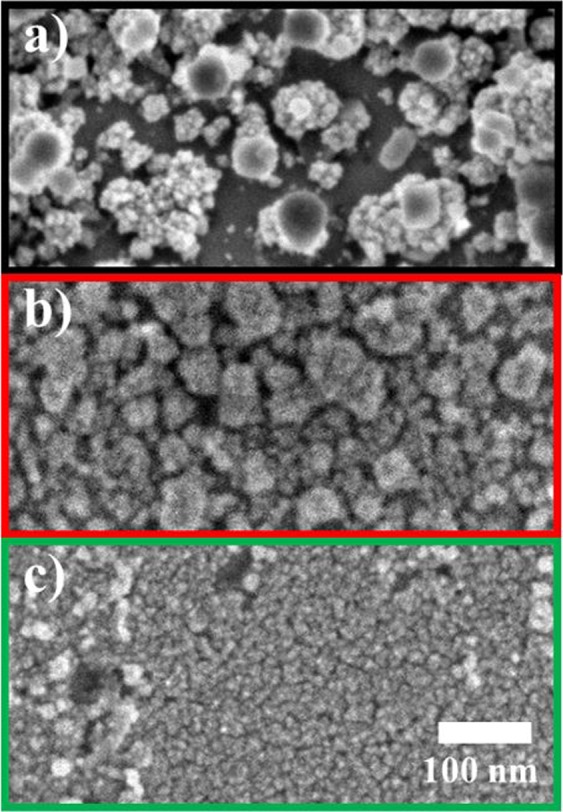
SEM images of Pt deposits on Sn, prepared by electrodeposition in borate buffer (pH = 8.4, 0.05 M) at (**a**) −0.10, (**b**) −0.15 and (**c**) −0.25 V_RHE_ for 204 s, applying a rotation rate of 200 rpm, interrupted by a 3 s rotational pulse of 3000 rpm every 15 s.

This is then also reflected in the morphology of the resulting deposits, which is shown in the SEM images of the electrodes after the electrodeposition experiment (Fig. 5a–c). In general, the electrodes are composed of a variety of small, well-connected Pt deposits with an island-like morphology. The lateral size of the deposits varies with respect to the applied deposition potential in the order −0.1 > −0.15 > −0.25 V_RHE_. The average island diameter at the most cathodic deposition potential of −0.25 V_RHE_ (Fig. 5c) is on the order of 10 nm, while deposits produced at −0.15 and −0.1 V_RHE_ have slightly larger dimensions. We conclude that the higher overpotential with respect to the HER on Pt at more cathodic potentials, causes the evolution of large amounts of H_2_ on the surface of the Pt deposits. This ongoing reaction shields the Pt deposits from successive reduction of ions on their surface, while pure Sn sites are more exposed to the solution. Hence, Pt deposition on Sn is favored over the deposition on the Pt islands and smaller deposits are formed. This, however, implies that the average film thickness would not grow significantly during longer potential application, since most of the charge in the plateau phase would correspond to HER as soon as a high coverage on the electrode is achieved. It shall be mentioned at this point, that the effect of the high H_2_ concentration in the vicinity of the electrode on the chemical stability of the PtCl_4_^2−^complex was not studied here and a certain amount of chemical deposition due to reduction of Pt ions by H_2_ cannot be fully excluded.

**Figure 6:**
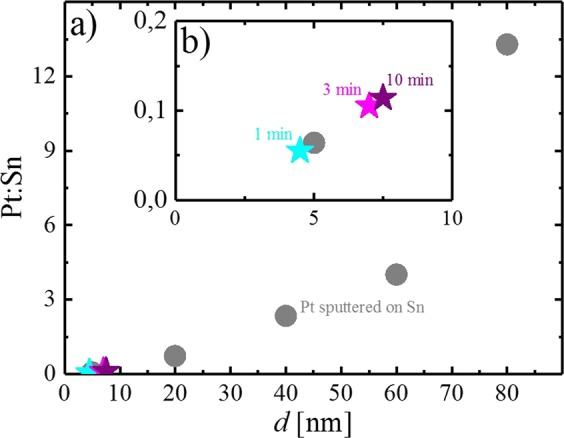
Ratio of the Pt:Sn counts vs. the equivalent thickness, *d*, of the Pt deposit on Sn. The counts were obtained by EDX analysis at 15 kV. The Pt layer was either prepared by sputter deposition (grey circles) or by electrochemical deposition for 1 (turquoise star), 3 (magenta star) or 10 min (purple star). While the full range of prepared samples is shown in a), the inset b) represents a zoom below 10 nm. Origin 9.1 (https://www.originlab.com).

To further investigate the Pt deposition efficiency during the plateau phase, electrochemical deposition using the same procedure employed earlier was carried out at a constant potential of −0.25 V_RHE_, varying the time from 68, 190 to 626 s (termed 1, 3 and 10 min hereafter). While the shortest deposition time is still in the region where the current increases, the other two deposition times were chosen such that the current plateau was reached, remaining at these conditions for a different amount of time. The equivalent thickness of the resulting deposits was analyzed by EDX, where the electron beam penetrates through the Pt surface layer into the Sn metal. Therefore, the ratio of the X-rays emitted by Pt and Sn contains intrinsic information about the thickness of the Pt overlayer. The Pt layer thickness was estimated by comparing the Pt:Sn count ratio to those obtained from samples of defined Pt layer thickness on Sn, obtained by sputter deposition. The thickness of the sputter-deposited Pt films was controlled using a quartz crystal microbalance (QCMB) in the deposition chamber. As expected, the Pt:Sn ratio of the sputter-deposited samples increases with nominal Pt layer thickness, as shown in Fig. 6 (grey circles). While the increase is nearly linear for thin Pt layers, a strong increase is observed for thicker layers (e.g., Pt:Sn ≈ 13 for 80 nm), which we hypothesize to originate from the lower beam penetration through the Pt layer as the thickness increases. Nevertheless, the general correlation of the Pt:Sn count ratio and the Pt overlayer thickness can be used to estimate the thickness of the Pt deposits on Sn, which have a comparably small Pt:Sn ratio of 0.055, 0.105 and 0.114 for 1, 3 and 10 min of electrodeposition, shown in the inset of Fig. 6 (turquoise, magenta and purple stars). The obtained values correspond approximately to a thickness of 4 nm after 1 min and 7–8 nm after 3 and 10 min. We therefore conclude that the efficiency of Pt deposition is highest in the initial phase, where most of the Sn surface sites are available. On the other hand, the deposition process slows down significantly in the plateau phase, where a large fraction of the current originates from HER on the Pt deposits, shielding the electrode surface.

### Electrochemical analysis of Pt deposits on Sn

**Figure 7:**
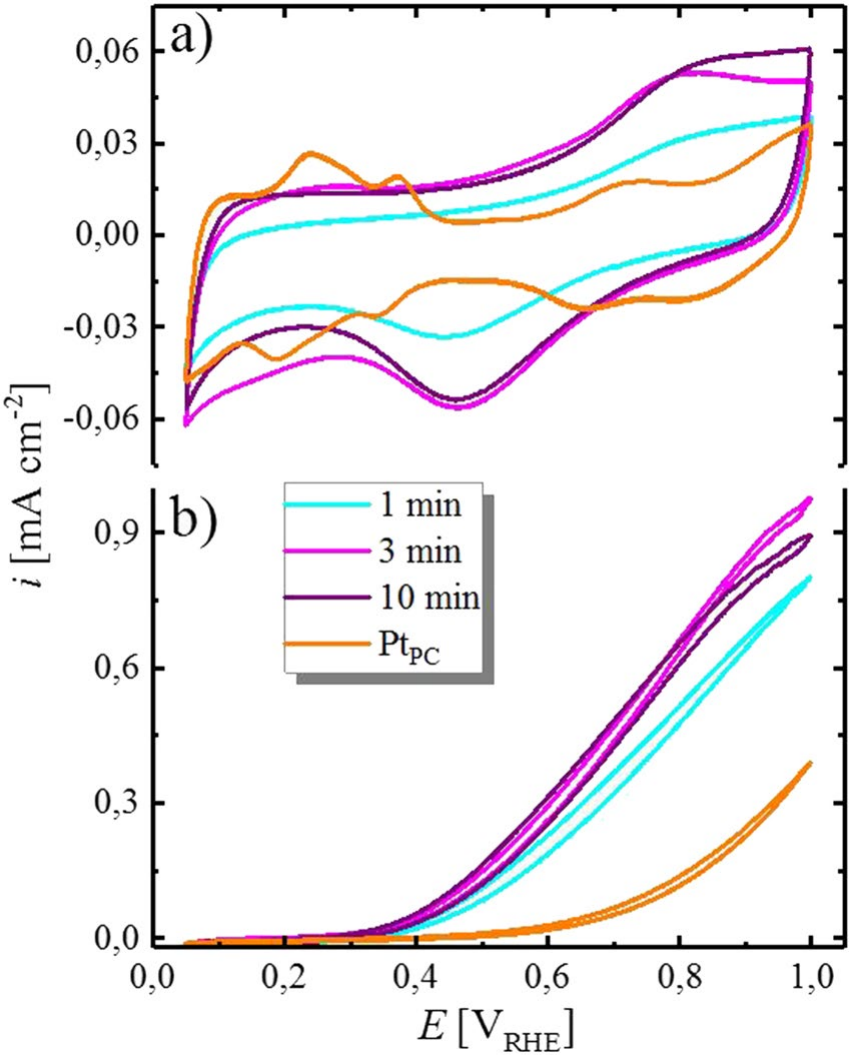
(**a**) CVs of electrochemically deposited Pt on Sn after a deposition for 1 (turquoise line), 3 (magenta line) and 10 min (purple line) at −0.25 V_RHE_ in Ar-saturated borate buffer (0.05 M, pH = 8.4) at a scan rate of 20 mV s^−1^ between 0.05 and 1.00 V_RHE_, measured in stagnant electrolyte at room temperature. The CV of a polycrystalline Pt disk is shown as comparison (orange line). (**b**) CVs of the same deposits after saturating the electrolyte with CO and scanning at 10 mV s^−1^ and a rotation rate of 400 rpm. Origin 9.1 (https://www.originlab.com).

Since the preceding EDX analysis showed that the approximate thickness of the deposits does not increase significantly during the current plateau phase, the analysis of this effect was complemented by measuring CVs of the electrodeposits in borate buffer solution, provided in Fig. 7a. First, it has to be noted that the general shape of the CV for all prepared deposits is similar, whereas the oxidative, as well as the reductive charge of the sample prepared at 1 min is significantly lower compared to 3 and 10 min. Furthermore, the charge of the CV of the electrodeposit after 3 min compares well with that after 10 min deposition time, indicating a similar exposed surface area. Since the main deposition time for both samples (3 and 10 min) was passed during the plateau phase, the previously stated hypothesis that the deposition during this phase is strongly limited, while the major fraction of the charge originates from HER, is hereby confirmed. Moreover, the charge of the CVs in the oxide formation/removal region (*E* > 0.4 V_RHE_) after Pt electrodeposition on Sn is higher compared to a bare Pt_PC_ disk. This is indicative of a high roughness factor (*rf*) of the Pt deposit on Sn, stemming from the highly structured surface compared to the flat, polished Pt electrode (*rf* ≈ 1.3, extracted from the averaged H_upd_ charge in the anodic and cathodic scan). Furthermore, the electrochemical features on the Pt electrodeposits are very different from pure Pt_PC_ (orange line), especially at low potentials, where no distinct adsorption features for the H_upd_ are present on the electrodeposits. Additionally, the oxidation and reduction potential of the Pt electrodeposit is further separated compared to pure Pt, indicating a larger required overpotential for this process. The differences of the CVs compared to pure Pt can be understood by considering the presence of Sn in the vicinity of Pt. In fact, Pt-Sn alloys are known to exhibit distinct electrochemical features compared to Pt, visible e.g., in acidic electrolyte^31,32^. This can also be observed in the CVs shown in Fig. 7a, where the oxidative and reductive features of the PtSn catalysts extend over a wider potential range compared to pure Pt (orange line) and the features in the potential window of the H_upd_ are not clearly resolved. Since such alloys are furthermore well-known to be excellent catalysts for the oxidation of carbon monoxide^33,34^, the electrolyte was saturated with CO, reporting steady-state CVs in Fig. 7b in order to probe the deposits for a possible alloy formation. Compared to Pt_PC_ (≈0.6 V_RHE_), the onset potential of the oxidation of CO on the Pt deposits on Sn is shifted approximately 200 mV cathodic, which is comparable to the results presented in the literature for PtSn alloys^34^. Additionally, depositing Pt for 1 min results in a slightly lower activity towards the CO oxidation, which might be mainly related to the incomplete coverage of Pt on the Sn surface, hence a lower *rf* value, as indicated by the CV in Fig. 7a. Therefore, the formation of a Pt-Sn alloy upon electrochemical deposition from borate buffer solution was confirmed by the CVs and CO oxidation experiments.

**Figure 8:**
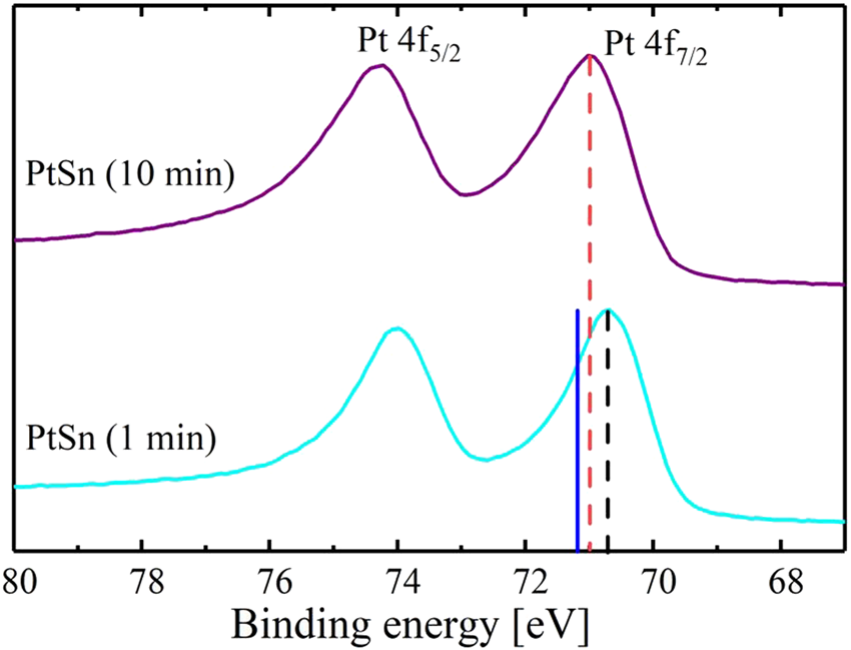
XPS spectra of the Pt 4 f region of PtSn samples after 1 min (turquoise line) and after 10 min (purple line) of electrochemical deposition. The blue solid line represents the Pt 4f_7/2_ binding energy for a pure Pt sample reported in the literature^35–37^. Origin 9.1 (https://www.originlab.com) and CasaXPS version 2.3.19PR1.0 (http://www.casaxps.com/).

To further confirm the formation of the PtSn surface alloy, XPS was used to analyze the Sn samples that were exposed to 1 min and 10 min of Pt deposition. Figure 8 shows the spectra of the Pt 4 f level for the two PtSn samples obtained at different deposition times (1 min and 10 min). The photoelectron peaks of the PtSn samples show a shift towards lower biding energies when compared to a pure Pt reference obtained from literature^35–37^. Moreover, the Sn ample on which Pt was deposited for 1 min shows the largest shift compared to the Pt reference, with a deviation of 0.5 eV. The nature of this shift can be explained by the formation of an alloy between the deposited Pt and Sn on the surface of the electrode^35,36^. On the other hand, the sample on which Pt was deposited for 10 min shows a smaller shift from the pure Pt reference, suggesting that the alloying between Pt and Sn is most likely diluted by pure Pt that couldn’t reach the Sn surface.

## Conclusions

Herein, we report insights into the electrochemical deposition process of Pt on Sn from a pH neutral borate buffer solution. Under the tested conditions, Pt electrodeposition was fully prevented when the Sn surface was covered with an oxide layer, even though high cathodic overpotentials were applied. In addition, the same general principle of self-limitation was found to be valid for Pt deposition on a Pt electrode and for a Pt covered Au surface. We conclude from these findings that the concept of surface oxide blocking, introduced here for the first time, has potential to be further developed into a controllable self-limiting deposition process. Such a process could, e.g., be based on the deposition of a Pt-ML on a metallic surface, thereafter oxidizing the surface of the Pt deposit and stopping further deposition.

Furthermore, a method was developed to deposit Pt on Sn in the absence of surface oxide, while HER proceeded on the Pt deposits. In this study, the effect of the applied deposition potential and -time on the resulting morphology were investigated. After an initiation period of a constant potential deposition, where the largest fraction of the Sn surface was covered with Pt, the deposition process was found to be self-limiting due to ongoing HER on Pt. Hence, increasing the deposition time resulted essentially in the same deposited layer thickness (<10 nm) and similar electrochemical behavior of the electrodes. Deposited Pt was found to alloy immediately with the Sn substrate, providing a high activity towards the oxidation of CO. In conclusion, the deposition procedure presented here can be used to reliably obtain Pt overlayers in the nanometer range, alloyed with the substrate in a single step.
